# Supplementary material for: High-resolution silkworm pan-genome provides genetic insights into artificial selection and ecological adaptation
Source: Nat Commun. 2022 Sep 24;13:5619. doi: 10.1038/s41467-022-33366-x (PMC9509368; doi:10.1038/s41467-022-33366-x)
Supplement: Supplementary file 1 — Supplementary Information [file 41467_2022_33366_MOESM1_ESM.pdf]

Supplementary Information

High-resolution silkworm pan-genome provides genetic insights into artificial selection and ecological adaptation

Tong X. *et al*.

This file includes Supplementary Figs. 1 to 7

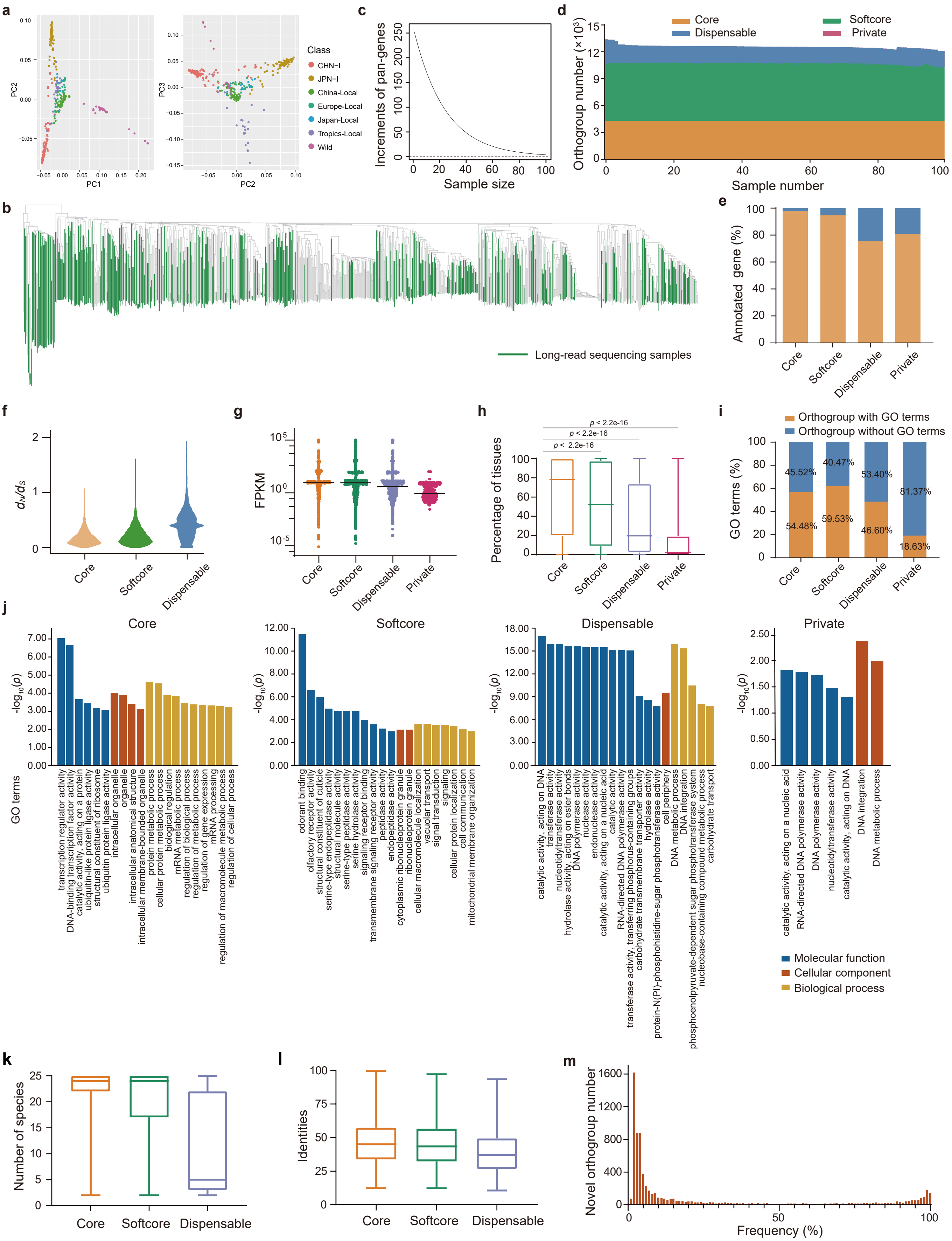

Supplementary Fig. 1 | Pan-gene analysis of 100 silkworm genomes. a, PCA of seven silkworm populations. **b,** Phylogenetic tree of the samples (545) used to perform long-read sequencing (green). **c,** Evaluation of pan-gene plateau. Derivative curve of fitted curve. **d,** The counts of core, softcore,

dispensable, and private gene clusters in each of the 100 genomes. **e**, Proportion of genes with (orange) and without (blue) InterPro domains annotation in the four clusters. **f**, d_N/d_S values of genes in core, softcore, and dispensable groups. **g** and **h,** The expression of core, softcore, dispensable, and private genes. Core genes expressed at a higher level (h) and in more tissues (i) than dispensable and private genes. Student's *t*-test (two-tailed). **i**, The percentages of core, softcore, dispensable, and private genes with and without GO annotation. **j**, Top 20 of GO enrichment terms of core, softcore, dispensable, and private genes. Silkworm core genes show the widest distribution (**k**) and the highest sequence identity (**l**) among 24 insects in 10 orders. **m**, Frequency distribution of newly identified genes in the 100 annotated genomes. Horizontal lines within boxes indicate the medians, box boundaries indicate the 1st and 3rd quartiles, and whiskers indicate the minima and maxima.

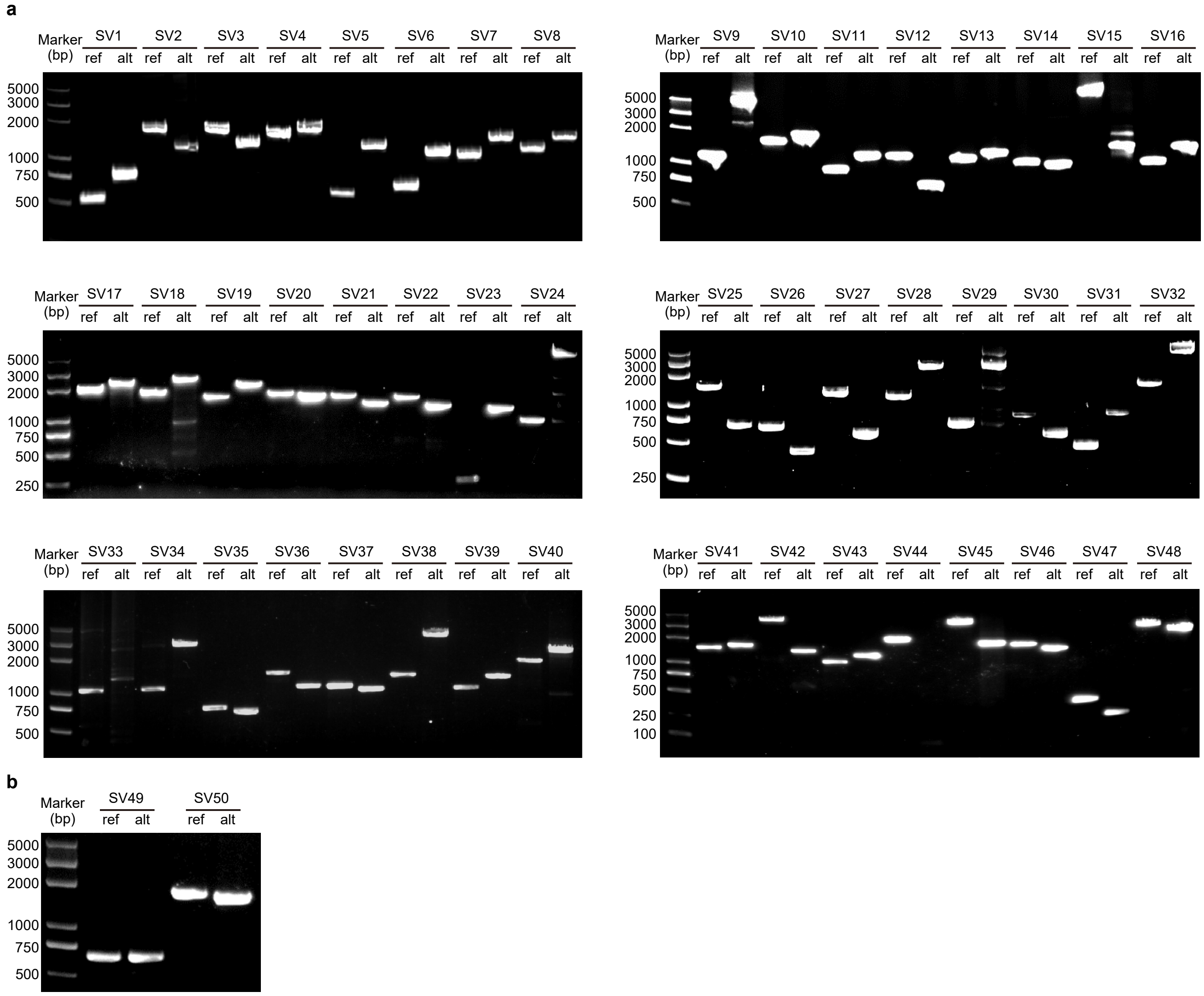

Supplementary Fig. 2 | SV verification. We randomly selected 50 SVs from the non-redundant SV set to evaluate accuracy of SV calling. We amplified those SVs using PCR and show DNA bands using agarose gel electrophoresis. **a**, 48 SVs verified as positive calling. **b**, Two SVs confirmed as negative calling. “ref” and “alt” represent reference genome and altered genome. The experiment was repeated twice with consistent results. Source figures are provided as a Source Data file.

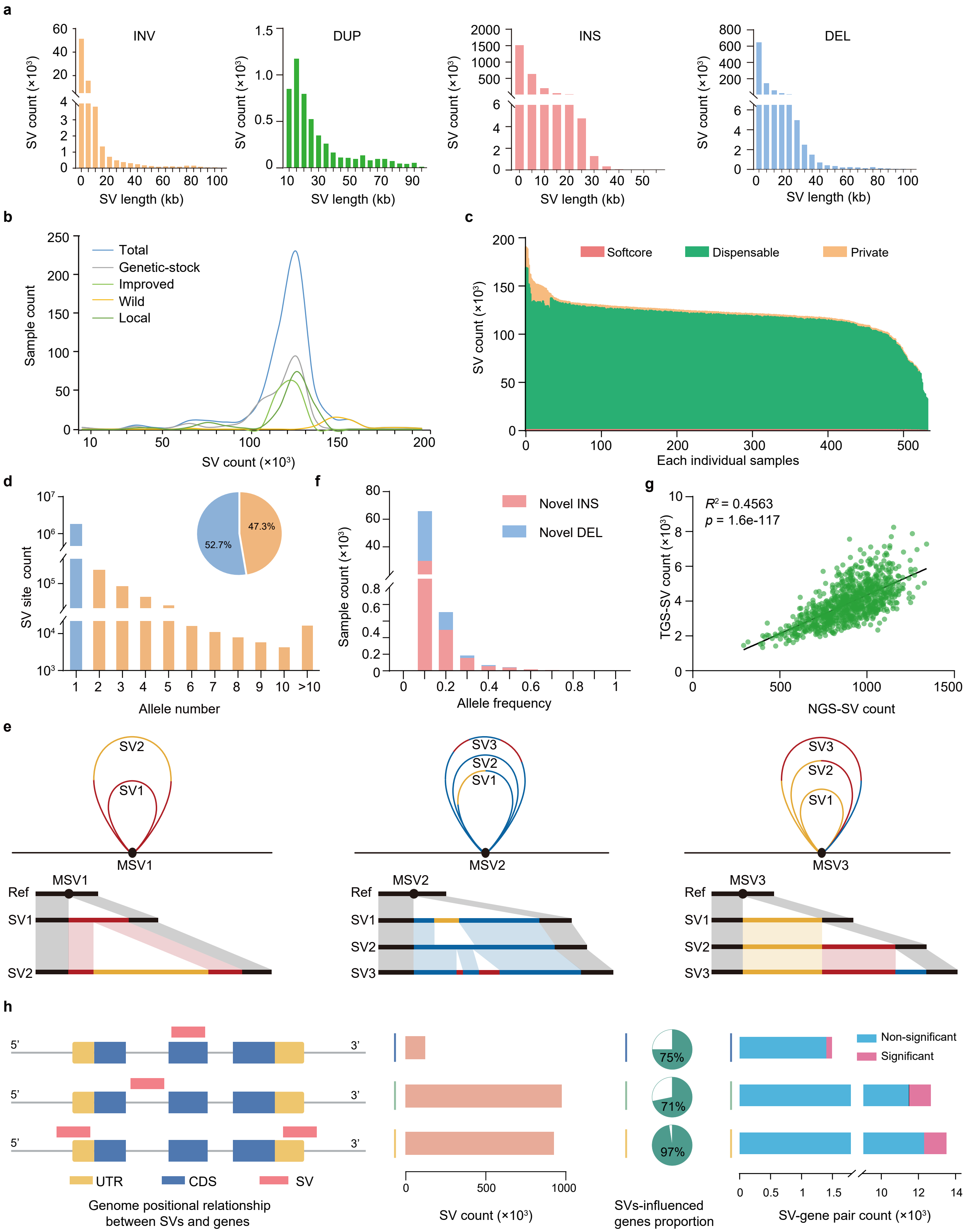

Supplementary Fig. 3 | Characterization of pan-SVs in 545 silkworm genomes. a, The distribution of insertion (INS), deletion (DEL), inversion (INV), and duplication (DUP) lengths. **b**, Line chart showing the sample count distribution with different SV count. **c**, The counts of softcore, private, and dispensable SVs for each of the 545 genomes. **d**, Single or multiple SV (MSV, ranging from 2 to 135) alleles found in

a certain genomic site. Histogram shows SV site counts with different allele numbers. The pie chart shows the proportions of SV sites with single (blue) and multiple (yellow) alleles. **e,** Schematic diagrams of genomic sites with MSV alleles and their collinearity. **f**, Allele frequencies of novel SVs newly identified in 537 NGS sequenced samples by mapping short reads against the graph-based pan-genome. **g**, Correlations of SV count distribution along chromosomes between TGS-SV and NGS-SV. The TGS-SVs were identified in the long-read sequenced genomes and the NGS-SVs were identified by mapping short reads of the 537 NGS sequenced samples to the graph-based pan-genome. SVs were counted on uninterrupted 500 kb windows along chromosomes. Linear regression, Pearson's r = 0.6755, $R^2 = 0.46$, p =1.6e-117, F-test, source data are provided as a Source Data file. **h**, SV impact on genes. SV count (middle) in the corresponding region (left) and their impact on gene expression (right histogram). The pie charts show the proportion of genes influenced by SVs.

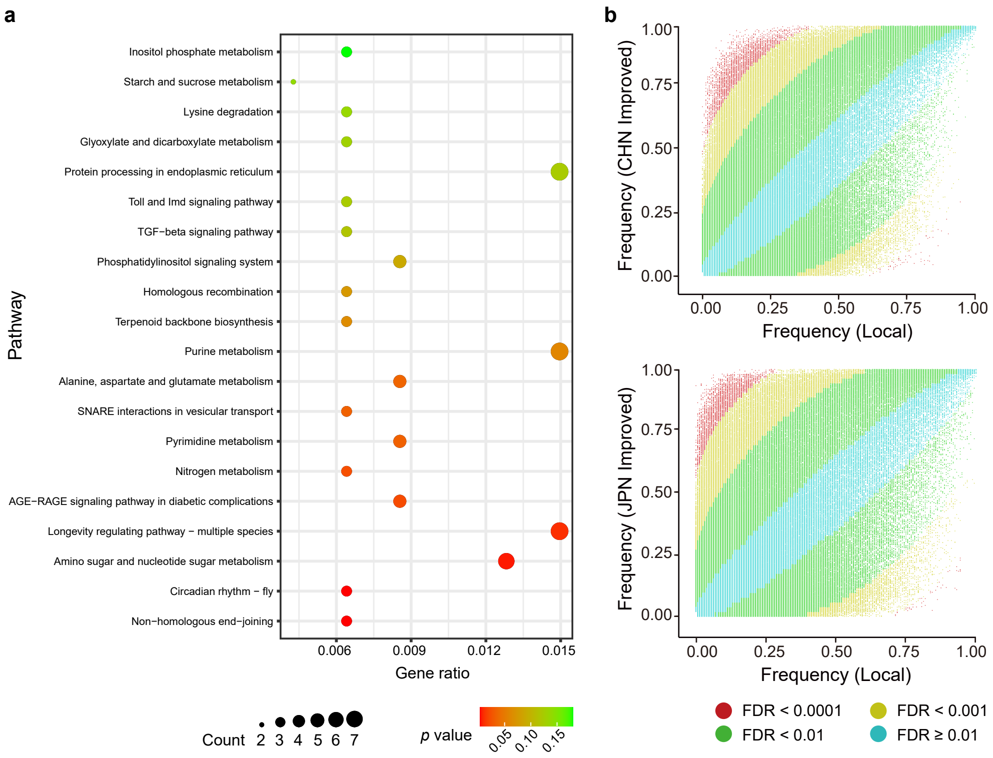

Supplementary Fig. 4 | KEGG enrichment of domestication-associated genes and SV frequencies in local and improved silkworms. a. KEGG pathway enrichment of domestication-associated genes. **b**. Allele frequencies of SVs in local and improved silkworms.

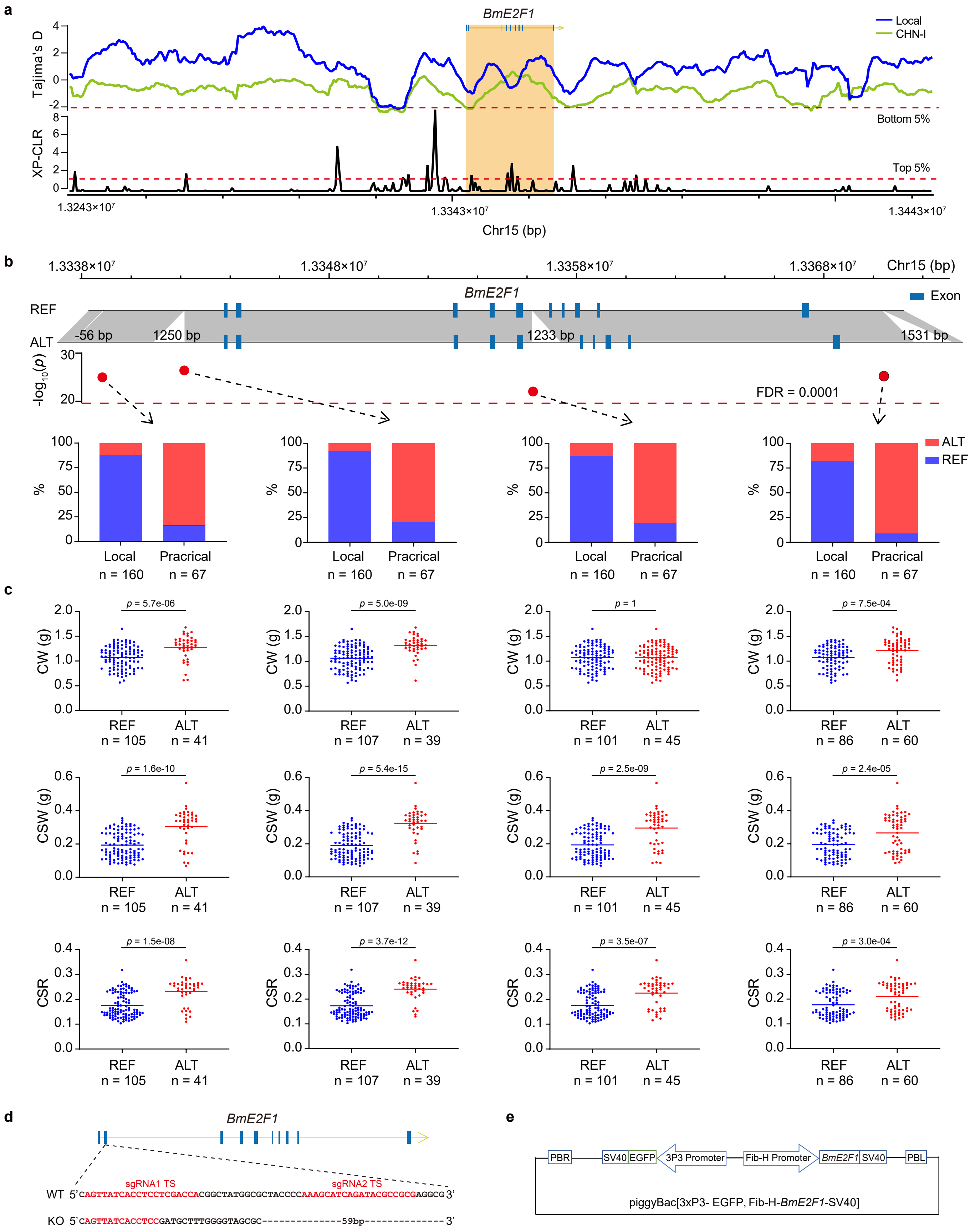

Supplementary Fig. 5 | Analysis of *BmE2F1* and relevant SVs. a, Signature of positive selection between local and CHN-I silkworms in the genomic region of the *BmE2F1* gene using Tajima's D and XP-CLR based selective sweep analysis. **b**, Four SVs with significant frequency divergence in the genomic region of *BmE2F1* between local and CHN-I silkworms. **c**, The cocoon weight (CW), cocoon

shell weight (CSW), and cocoon shell ratio (CSR) between strains with and without the four SVs. The line indicates the mean, Student's *t*-test (two-tailed). The line indicates the mean. **d**, CRISPR-cas9 mediated knockout of *BmE2F1*. **e**, Overexpression vector of *BmE2F1*. Source data are provided as a Source Data file.

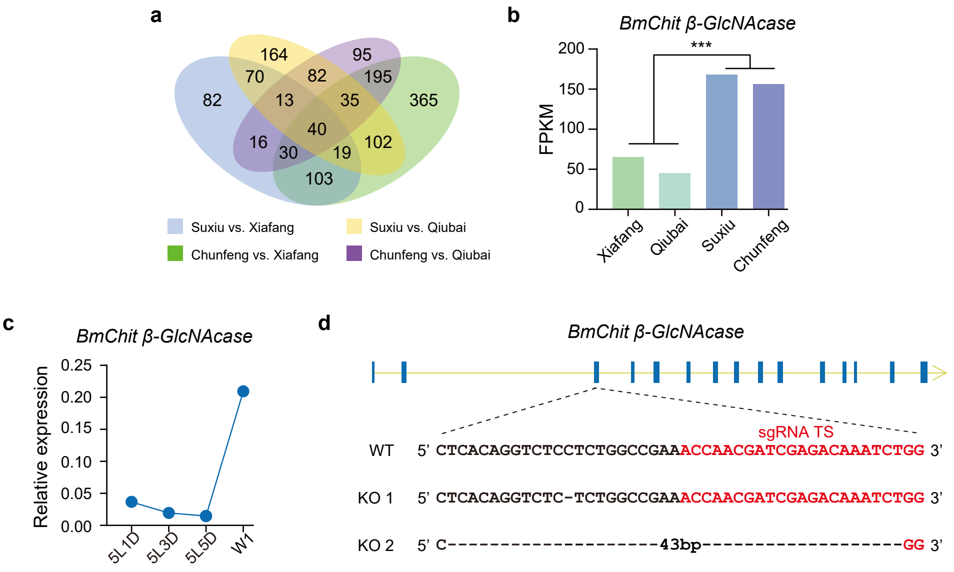

Supplementary Fig. 6 | Identification and analysis of a silk fineness related gene. a, Differential expressed genes (DEGs) between fine silk and coarse silk strains. **b**, The expression of *BmChit β-GlcNAcase* among the four strains. ***, $p = 0.0117$, Student's t-test (two-tailed). **c**, The expression profile of the *BmChit β-GlcNAcase* gene in silk press from the first day of fifth instar to the wandering stage (a stage at the start of spinning). **d**, CRISPR-cas9 mediated knockout of *BmChit β-GlcNAcase*. Source data are provided as a Source Data file.

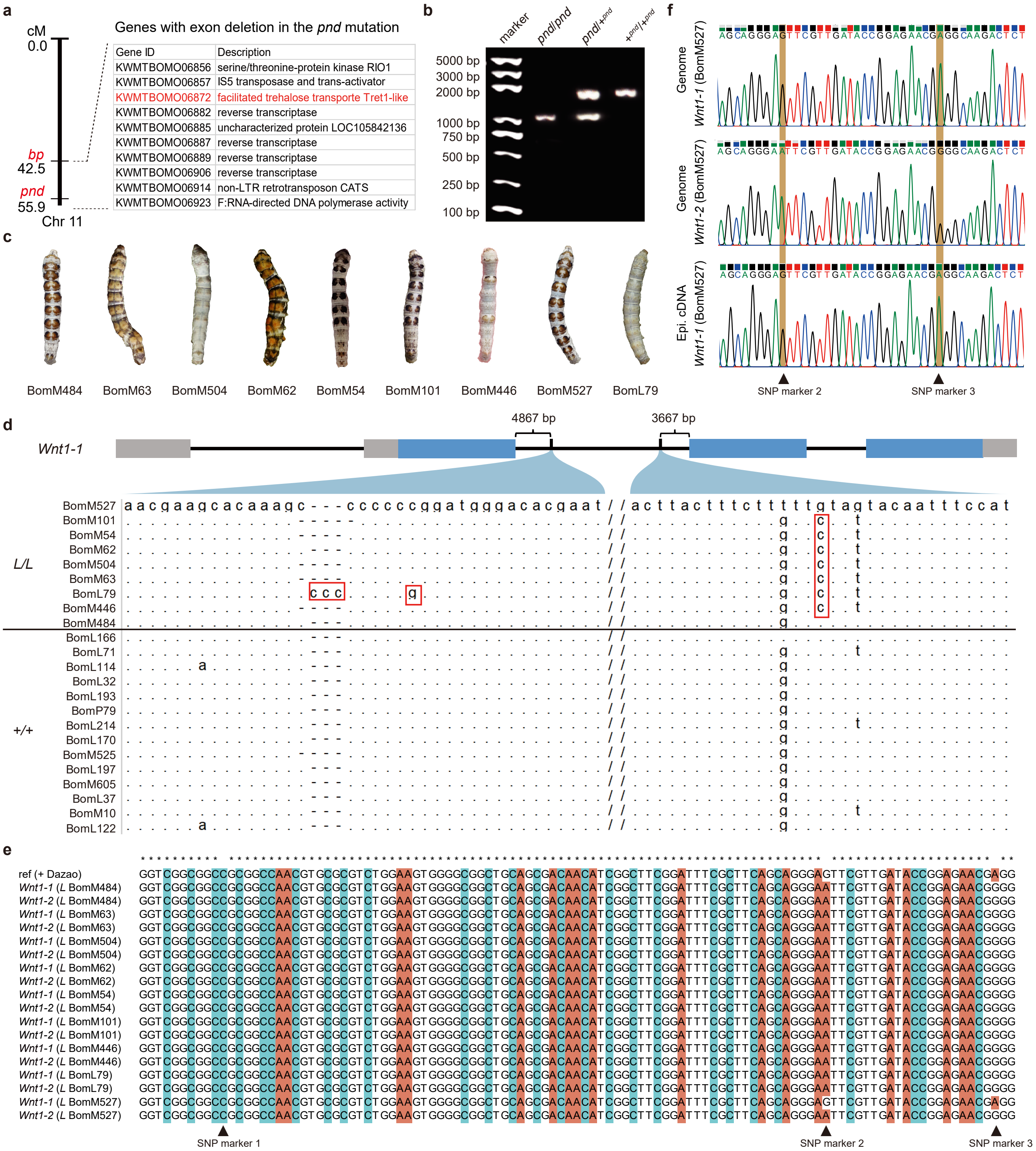

Supplementary Fig. 7 | The analysis of genes and variations in *pnd* and *L*. a, Genes with exonic variation in the *pnd* mutation. **b**, PCR gel image of *BmTret1-like* 3'UTR in *pnd*/*pnd*, *pnd*/+, and +/+. **c**, Phenotypes of nine *L* strains. The experiment was repeated twice with consistent results. Source figure is provided as a source data file. **d**, Variations in *Wnt1-1* gene region and its flanking 20 kb region. "." represents the same nucleotide as the first line, "-" represents gap or deletion in the corresponding position. The red boxes show the three variations that are specifically present but not fixed in the *L* strains. **e**, Multiple sequence alignment of *Wnt1*. The three black triangles indicate the three SNP markers previously identified in *Wnt1*. The *Wnt1-1* and *Wnt1-2* in *L* strain BomM527 are distinguished by SNP markers 2 and 3. **f**, Verification of two SNPs (markers 2 and 3) between *Wnt1-1* and *Wnt1-2* in the BomM527 genome using PCR and Sanger sequencing (based on the SNP waveform in the Sanger sequence). *Wnt1-1* is specifically expressed in the epidermis in the *L* strain (BomM527).
