# Supplementary material for: High-resolution silkworm pan-genome provides genetic insights into artificial selection and ecological adaptation
Source: Nat Commun. 2022 Sep 24;13:5619. doi: 10.1038/s41467-022-33366-x (PMC9509368; doi:10.1038/s41467-022-33366-x)
Supplement: Supplementary file 3 — Description of Additional Supplementary Files [file 41467_2022_33366_MOESM3_ESM.pdf]

Description of Additional Supplementary Files

File name: Supplementary Data 1
Description:
Information and NGS sequencing summary of the 1,078 samples

File name: Supplementary Data 2
Description:
Summary of ONT sequencing and structural variations of 545 genomes

File name: Supplementary Data 3
Description:
Genome assembly, annotation and pan-gene statistics of the 100 assembled genomes

File name: Supplementary Data 4
Description:
Comprehensive pan-genome sequence content and gene annotation

File name: Supplementary Data 5
Description:
List of the species used for investigating the similarity and distribution of core, softcore, and dispensable genes among insects

File name: Supplementary Data 6
Description:
Nine previously reported SVs were validated in this study

File name: Supplementary Data 7
Description:
The primer sequences used in the SV validation experiments

File name: Supplementary Data 8
Description:
8a: Genes and relevant SVs associated with silkworm domestication
8b: Genes and relevant SVs associated with silkworm improvement

File name: Supplementary Data 9
Description:
Lists of the primers, sgRNAs, and siRNAs used in this study
