# Supplementary material for: High-resolution silkworm pan-genome provides genetic insights into artificial selection and ecological adaptation
Source: Nat Commun. 2022 Sep 24;13:5619. doi: 10.1038/s41467-022-33366-x (PMC9509368; doi:10.1038/s41467-022-33366-x)
Supplement: Supplementary file 13 — Reporting Summary [file 41467_2022_33366_MOESM13_ESM.pdf]

nature portfolio

Corresponding author(s): Fangyin Dai, Zhonghuai Xiang, Wen Wang, Zhixi Tian, Cheng Lu, Xiaoling Tong

Last updated by author(s): Sep 8, 2022

# Reporting Summary

Nature Portfolio wishes to improve the reproducibility of the work that we publish. This form provides structure for consistency and transparency in reporting. For further information on Nature Portfolio policies, see our Editorial Policies and the Editorial Policy Checklist.

## Statistics

For all statistical analyses, confirm that the following items are present in the figure legend, table legend, main text, or Methods section.

| n/a | Confirmed | |
|---|---|---|
| ☐ | ☒ | The exact sample size (*n*) for each experimental group/condition, given as a discrete number and unit of measurement |
| ☒ | ☐ | A statement on whether measurements were taken from distinct samples or whether the same sample was measured repeatedly |
| ☐ | ☒ | The statistical test(s) used AND whether they are one- or two-sided<br>*Only common tests should be described solely by name; describe more complex techniques in the Methods section.* |
| ☒ | ☐ | A description of all covariates tested |
| ☐ | ☒ | A description of any assumptions or corrections, such as tests of normality and adjustment for multiple comparisons |
| ☒ | ☐ | A full description of the statistical parameters including central tendency (e.g. means) or other basic estimates (e.g. regression coefficient) AND variation (e.g. standard deviation) or associated estimates of uncertainty (e.g. confidence intervals) |
| ☐ | ☒ | For null hypothesis testing, the test statistic (e.g. *F*, *t*, *r*) with confidence intervals, effect sizes, degrees of freedom and *P* value noted<br>*Give P values as exact values whenever suitable.* |
| ☒ | ☐ | For Bayesian analysis, information on the choice of priors and Markov chain Monte Carlo settings |
| ☒ | ☐ | For hierarchical and complex designs, identification of the appropriate level for tests and full reporting of outcomes |
| ☒ | ☐ | Estimates of effect sizes (e.g. Cohen's *d*, Pearson's *r*), indicating how they were calculated |

*Our web collection on statistics for biologists contains articles on many of the points above.*

## Software and code

Policy information about availability of computer code

| | |
|---|---|
| Data collection | no software was used for data collection |
| Data analysis | In this study, a total of 55 previous softwares were used, including SOAPnuke (v1.5.6), guppy_barcoder (v3.1.50), qcat (v1.0.1), porechop (v0.2.4), BWA (v0.7.170), SAMtools (v1.11), Picard (v2.23.5), GATK (v4.1.8.1), VCF2Dis (v1.42), PHYLIPNEW (v3.69), plink(v2.0),GCTA(v1.93.0), jellyfish (v2.2.6), genomeScope (v1.0), Canu (v1.8), Smartdenovo (v1.0), racon (v1.3.3), medaka (v0.7.1), minimap2 (v2.17), pilon (v1.23), Purge Haplotigs (v1.0.0), RagTag (v1.0.0), BUSCO (v5.2.1), GMATA (v2.2), TRF (v4.09), MITE-Hunter (v2), LTR_finder (v1.07), GenomeTools (v1.5.9), LTR_retriver (v2.9.0), RepeatModeler (v2.0.1), TEclass (v2.1.3c), RepeatMasker (v4.1.1), GeMoMa (v1.6.10), STAR (v2.7.3a), Stringtie (v2.1.4), PASA (v2.3.3), augustus (v3.4.0), EVidenceModeler (v1.1.1), InterProScan (v5.41-82.0), BLAST+ (v2.9.0), tRNAscan-SE (v2.0), Infernal (v1.1.3), RNAmmer (v1.2), OrthoFinder (v2.3.7), NGMLR (v0.2.7), Sniffles (v1.0.11), SURVIVOR (v1.0.3), Jasmine (v1.0.1), RMBlast (v2.9.0-p2), Vcfanno (v0.3.2), Vg toolkit (v1.30.0), bowtie2 (v2.4.2), RSEM (v1.3.3), CRISPRdirect(v140413), R (v4.0.3). |

For manuscripts utilizing custom algorithms or software that are central to the research but not yet described in published literature, software must be made available to editors and reviewers. We strongly encourage code deposition in a community repository (e.g. GitHub). See the Nature Portfolio guidelines for submitting code & software for further information.

## Data

Policy information about availability of data

All manuscripts must include a data availability statement. This statement should provide the following information, where applicable:

- Accession codes, unique identifiers, or web links for publicly available datasets
- A description of any restrictions on data availability
- For clinical datasets or third party data, please ensure that the statement adheres to our policy

All long-read, short-read, and RNA sequencing data from this study have been deposited into the CNGB Nucleotide Sequence Archive (CNSA) of China National GeneBank DataBase (CNGBdb, https://db.cngb.org) with BioProject: CNP0001815 (https://db.cngb.org/search/project/CNP0001815/) and also into the Genome Sequence Archive (https://ngdc.cncb.ac.cn/gsa/) with accession number CRA007878 (https://bigd.big.ac.cn/gsa/browse/CRA007878). All 545 genome assemblies, 100 genome annotations (gff files), pan-genome, and VCF files (SNP, SV) were also deposited in the CNGBdb with BioProject ID CNP0002456 (https://db.cngb.org/search/project/CNP0002456/). This study also analyzed data for four wild silkworm genomes that are available in the Sequence Read Archive (SRA) database according to accession numbers DRX054041(https://www.ncbi.nlm.nih.gov/sra/DRX054041), DRX054040 (https://www.ncbi.nlm.nih.gov/sra/DRX054040), ERS402904 (https://www.ncbi.nlm.nih.gov/sra/ERS402904), ERS402902 (https://www.ncbi.nlm.nih.gov/sra/ERS402902).

## Human research participants

Policy information about studies involving human research participants and Sex and Gender in Research.

| | |
|---|---|
| Reporting on sex and gender | N/A |
| Population characteristics | N/A |
| Recruitment | N/A |
| Ethics oversight | N/A |

Note that full information on the approval of the study protocol must also be provided in the manuscript.

# Field-specific reporting

Please select the one below that is the best fit for your research. If you are not sure, read the appropriate sections before making your selection.

☒ Life sciences ☐ Behavioural & social sciences ☐ Ecological, evolutionary & environmental sciences

For a reference copy of the document with all sections, see nature.com/documents/nr-reporting-summary-flat.pdf

# Life sciences study design

All studies must disclose on these points even when the disclosure is negative.

| | |
|---|---|
| Sample size | A total of 1078 silkworms, containing 205 local strains, 194 improved varieties, 632 genetic stocks, and 47 wild silkworms, were sequenced by next-generation sequencing technology. These 1,078 genomes represent almost entire pan-genome of silkworm. The local and wild silkworms were collected from a full range of their geographic distribution. Among these genomes, 545 representative silkworms were selected based on phylogenetic relationship and represented genetic diversity in silkworms. |
| Data exclusions | No data was excluded from the analyses |
| Replication | Three biological replicates with three technical replicates were used in the qRT-PCR experiment. All replications were successful and were used. |
| Randomization | This was an observation study, there was no randomization. |
| Blinding | Blinding does not apply to this study. Blinding is not necessary because this study focuses on comparative genomics. |

# Reporting for specific materials, systems and methods

We require information from authors about some types of materials, experimental systems and methods used in many studies. Here, indicate whether each material, system or method listed is relevant to your study. If you are not sure if a list item applies to your research, read the appropriate section before selecting a response.

## Materials & experimental systems

| n/a | Involved in the study |
| --- | --- |
| ☒ | ☐ Antibodies |
| ☒ | ☐ Eukaryotic cell lines |
| ☒ | ☐ Palaeontology and archaeology |
| ☐ | ☒ Animals and other organisms |
| ☒ | ☐ Clinical data |
| ☒ | ☐ Dual use research of concern |

## Methods

| n/a | Involved in the study |
| --- | --- |
| ☒ | ☐ ChIP-seq |
| ☒ | ☐ Flow cytometry |
| ☒ | ☐ MRI-based neuroimaging |

# Animals and other research organisms

Policy information about studies involving animals; ARRIVE guidelines recommended for reporting animal research, and Sex and Gender in Research

| | |
| --- | --- |
| Laboratory animals | The study did not involve laboratory animals. |
| Wild animals | Larvae of 47 wild silkworms were captured from a full range of their geographic distributions in China. Sex and capture place were described in Supplementary Data 1. These wild silkworms are transported in live form. These wild silkworm larvae are then raised in the laboratory until they developed into pupa. The pupa were stored in the liquid nitrogen and were transported to the sequencing company. Since the wild silkworm is very small, only the entire pupa can extract the amount of DNA required for genome sequencing. So these wild silkworms were killed after the study. |
| Reporting on sex | No findings applied on only one sex. |
| Field-collected samples | The wild silkworms were collected the field that was described in Supplementary Data 1, but experiments were not performed in the field, as terminal samples were collected. |
| Ethics oversight | No ethical approval or guidance was required because silkworm did not involve ethical problem. |

Note that full information on the approval of the study protocol must also be provided in the manuscript.
